# Limit Theorems as Blessing of Dimensionality: Neural-Oriented Overview

**DOI:** 10.3390/e23050501

**Published:** 2021-04-22

**Authors:** Vladik Kreinovich, Olga Kosheleva

**Affiliations:** Departments of Computer Science (V.K.) and Teacher Education (O.K.), University of Texas at El Paso, El Paso, TX 79968, USA; olgak@utep.edu

**Keywords:** limit theorems, curse and blessing of dimensionality, neural networks

## Abstract

As a system becomes more complex, at first, its description and analysis becomes more complicated. However, a further increase in the system’s complexity often makes this analysis simpler. A classical example is Central Limit Theorem: when we have a few independent sources of uncertainty, the resulting uncertainty is very difficult to describe, but as the number of such sources increases, the resulting distribution gets close to an easy-to-analyze normal one—and indeed, normal distributions are ubiquitous. We show that such limit theorems often make analysis of complex systems easier—i.e., lead to blessing of dimensionality phenomenon—for all the aspects of these systems: the corresponding transformation, the system’s uncertainty, and the desired result of the system’s analysis.

## 1. Introduction: From Curse of Dimensionality to Blessing of Dimensionality

### 1.1. First, a Curse

Often, the more we analyze a system, the more accurately we want to predict its behavior—the more factors we need to take into account, the more complex the system’s behavior.

In some cases, real-life data is intrinsically low-dimensional: most of the factors can be reduced to a few of them. However, in many other real-life situations, all these factors are important. As a result, as a system’s description becomes more complex, analyzing this system becomes more complicated. This phenomenon is known as a *curse of dimensionality*.

### 1.2. Then, a Blessing

Interestingly, often, a further increase in the system’s complexity often makes this analysis simpler. Following [1], we will call this phenomenon a *blessing of dimensionality*.

### 1.3. Example

A classical example of this first-curse-then-blessing phenomenon is the joint effect of many random phenomena. When we know the probability distribution of each phenomenon, in principle, we can compute their joint effect—but, as the number of these phenomena becomes larger and larger, the corresponding computations become more and more complicated. At first glance, this is a classical example of the curse of dimensionality.

However, as the number of these phenomena increases further, we start seeing the effect of the Central Limit Theorem (see, e.g., [2]), according to which, under reasonable conditions, the joint effect of many small independent random phenomena is close to Gaussian. The resulting distribution becomes very close to the easy-to-analyze Gaussian distribution—and this is one of the main reasons why normal (=Gaussian) distributions are ubiquitous.

### 1.4. Other Examples

In the last decade, many other examples of blessing-of-dimensionality appeared, both in the general analysis of complex systems (see, e.g., [1,3,4,5,6,7]) and, specifically, in the analysis of neural networks; see, e.g., [8,9,10,11].

### 1.5. Are These Lucky Examples or a General Trend?

At first glance, it may appear that all these examples are lucky breaks in the dark world of curse-of-dimensionality phenomena. So, a natural question is: is this pessimistic viewpoint correct—or blessing-of-dimensionality results are ubiquitous?

### 1.6. It Is a General Trend

In this paper, we show that the above pessimistic viewpoint is unnecessarily pessimistic. Actually, as we will show, similar limit theorems are ubiquitous—and their use can (and do) help in data processing in general—and, in particular, when using neural networks to process data.

While most above-cited blessing of dimensionality results are related to a statistical description of some phenomenon, we show that there are other limit theorems that are related to non-random phenomena.

We also show that limit theorems help explain the surprising empirical success of many techniques, from traditional neural networks to convex techniques and clustering.

### 1.7. Caution: Blessing-of-Dimensionality Is Not a Panacea

- The fact that limit theorems can explain some empirical successes does not mean, of course, that these blessing-of-dimensionality results are the only reason for these empirical successes: sometimes, as we have mentioned, the multi-dimensional data is actually intrinsically low-dimensional.
- The fact that limit theorems *often* make data processing easier does not mean that as the data complexity increases, the analysis *always* becomes simpler: many problems remain complex. At present, there is no clear general understanding of when the blessing of dimensionality occurs and where it does not occur. It would be nice to find such an understanding.

### 1.8. What We Do in This Paper

In this paper, we review, in an expository mathematics format, several published results (some of them our own) showing that limit theorems can simplify the analysis of complex systems in general and neural networks in particular.

Our main interest is in applications to neural networks, so when a theorem has such applications, we explicitly mention them—but we mention other applications as well. The number of neural applications of limit theorems is, at present, not large, but we hope that papers like this one—which explain how such theorems are successfully used in other applications–will encourage interested readers to develop new applications of these blessing-of-dimensionality results to neural networks.

The intended audience of this paper are readers with a conceptual understanding of the mathematics involved, not necessarily with a specialist knowledge. Readers interested in more detailed discussions and/or exact formulations and proofs are welcome to look at the corresponding papers listed in the bibliography. In these papers, the corresponding discussions, formulations, and proofs are presented in all necessary detail.

The general study of blessing-of-dimensionality phenomena has started only a few decades ago, there are still more open problems than results—and available results are mostly breakthroughs in different directions, not yet forming a very coherent picture. Good news is that there are already many such results, and their applications already over many areas. We hope that by listing these results and some of their applications, we will encourage interested readers to get involved in the related research—and that, together, we will make this phenomenon even more ubiquitous.

### 1.9. How This Paper Is Structured

We start, in Section 2, with classifying sources of dimensionality into spatial and temporal. Such a distinction is well known in neural network applications; in this section, we extend it to the general case of complex systems. Section 3 deals with spatial dimensionality, of which the dimensionality corresponding to the Central Limit Theorem is one of the examples. We start, in Section 3.1, with a new application of the Central Limit Theorem. In Section 3.2, we consider generalizations of Central Limit Theorem to other types of probability distributions. In Section 3.3, we consider limit theorems corresponding to the case when we do not know the corresponding probabilities, when we only know the set of possible values of the corresponding quantity or quantities. Section 3.4 lists related open questions. Finally, Section 4 deals with limit theorems related to temporal dimensionality.

## 2. Two Main Sources of Dimensionality: Spatial and Temporal

To provide an adequate analysis of the situation, let us first observe that in general, there are two main sources of dimensionality:

- First, at each moment of time, there is usually a large number of phenomena—located, in general, at different points in space—that need to be taken into account. Even if we use a few parameters to describe each of these phenomena, overall, we will need a very large number of parameters to describe all these phenomena—and thus, the dimensionality of the problems grows. We will call this dimensionality of *spatial origin*, or simply *spatial dimensionality*, for short. The above-mentioned Central Limit Theorem is a good example of spatial dimensionality.
- Furthermore, there may be parameters describing the history of the analyzed phenomenon—which also affect its current state. What naturally comes to mind is that the values of physical quantities change with time. In some cases, we observe these changes and we can analyze the corresponding time series. In other cases, we only observe the final results of these changes: e.g., inside a sensor, the original value may be transformed many times, and what we get as a resulting signal is the result of all these past transformations. In yet other cases, what changes are the simulated values—e.g., when we apply iterative algorithms. We will call the resulting dimensionality of *temporal origin*, or simply *temporal* dimensionality.

Furthermore, of course, in many real-life phenomena, we have both spatial and temporal sources of dimensionality which are difficult to separate. A neural-related example of such phenomena is traveling waves; see, e.g., [12,13].

In this paper, we will mention the limit theorems related to both spatial and temporal sources of dimensionality—and we hope that these results can be extended to the phenomena where both sources are intertwined.

*Comment.* Limit theorems are often somewhat complicated to understand and prove. In our experience, a better understanding of a complex multi-dimensional phenomenon is usually achieved if we consider easier-to-analyze few-dimensional particular cases or analogues. For limit theorems, a natural few-dimensional analogues are iterative methods in numerical mathematics, such as:

- Newton’s iterative method
  $$x^{\left(k+1\right)}=x^{\left(k\right)}-\frac{f\left(x^{\left(k\right)}\right)}{f^{\prime }\left(x^{\left(k\right)}\right)}$$
  for finding the solution to the equation f(x)=0 or
- the gradient descent method
  $$x_{i}^{\left(k+1\right)}=x_{i}^{\left(k\right)}-\alpha \cdot {\frac{\partial f}{\partial x_{i}}}_{|x=x^{\left(k\right)}}$$
  for finding the minimum of a function f(x); we mention this method, since back-propagation, the main way neural networks learn, is, from the mathematical viewpoint, exactly gradient descent—with additional computational simplifications; see, e.g., [14,15].

In both examples, convergence is not guaranteed, and the results explaining when there is convergence are often difficult to prove. However, what is much easier to prove is that *if* there is a convergence, then the limit satisfies the desired property—e.g., for Newton’s method, the limit value *x* satisfies the property f(x)=0. In some cases, the limit value only satisfies part of this desired property: for example, for the gradient descent method, the limit is always a stationary point, but not necessarily the desired global minimum of the objective function f(x). Indeed:

- For Newton’s method, if $x^{\left(k\right)}\to x$, then, in the limit, we get $x=x-\frac{f(x)}{f^{\prime }\left(x\right)}$, which implies that f(x)=0.
- For the gradient descent, if $x^{\left(k\right)}\to x$, then, in the limit, we get $x_{i}=x_{i}-\alpha \cdot \frac{\partial f}{\partial x_{i}}$, which implies that $\frac{\partial f}{\partial x_{i}}=0$. Thus, the limit point is always a stationary point, which is a necessary (but, as is well known, not sufficient) condition for it being the location of the minimum.

Similarly to these cases, in this paper, we will concentrate not so much on the conditions under which the processes converge, but rather on the description of the limit cases *when* there is convergence.

## 3. Dimensionality of Spatial Origin

As we have mentioned, the standard Central Limit Theorem is an example of what we called dimensionality of spatial origin. While many consequences of this theorem are well known, as we will show, there are many aspects of this theorem which still need exploring. So, the first thing we will consider—in the first subsection of this section—is what are the less known consequences of the Central Limit Theorem.

Of course, the limit distribution does not have to be normal: as we have mentioned, the convergence to the normal distribution happens only under certain conditions. For situations when these conditions are not satisfied, there are more general limit theorems. Applications of these more general theorems—mostly to uncertainty quantification—is what we will overview in the second subsection of this section.

All this assumes that we know the probability distributions that we are trying to combine. However, what if we do not know the probabilities, what if we only know the corresponding range of possible values—and we do not know the probabilities of different points from this range? This situation is discussed in the third subsection of this section.

This section ends with related open questions.

### 3.1. Not-Well-Known Consequences of the Central Limit Theorem

#### 3.1.1. Why Are Many Things in the World Discrete?

Outside quantum physics, most physical processes are continuous, most probability distributions are continuous—so what we should observe should be continuous as well. However, in reality, many things in the real world are discrete. We do not have weather continuously changing from sunny to rain: most of the time, we either have a sunny day or a rainy day. Yes, it is possible to have hybrid animals like mules, but most of the time, animals we see fall into one of the precise categories.

In many specific examples, there is a specific explanation for this discreteness—e.g., Darwin’s Theory of Evolution explains that only mutations which are beneficial to the individuum survive, and all intermediate stages between two beneficial states become extinct quickly. However, the very fact that the same discreteness phenomenon appears in many different application areas seems to be an indication that discreteness is a general phenomenon that must have a general explanation.

Discreteness is observed in machine learning as well: when we use a neural network (or any similar tool) for classification, what this network actually produces are continuous numbers that can be converted, e.g., to degrees to which the object belongs to different categories. However, usually, we do not return these degrees to the user. What we usually do at the end is select one of these categories (e.g., the most probable one)—and in most cases, this is exactly the desired classification, cat or dog, car or not-a-car, disease or healthy, and this is usually exactly what the users want.

This discreteness definitely helps when making decisions—instead of a continuum of possible values, we need to deal with only a few discrete ones. So, this discreteness can be viewed as an example of a blessing of dimensionality.

However, why are we mentioning this discreteness? At first glance, it may seem to be unrelated to the Central Limit Theorem—which is all about the normal distribution, which is, of course, absolutely continuous. Interestingly, there is a relation. Let us describe it.

#### 3.1.2. This Puzzling Discreteness Has Been Observed before

Of course, we are not the first ones who noticed that, in spite of the the fact that many processes are continuous, what we observe is often discrete. For example, B. S. Tsirelson noticed in [16] that in many cases, when we reconstruct a signal from noisy data, and we assume that the resulting signal belongs to a certain class, the reconstructed signal is often an *extreme* point from this class, i.e., it is one of the discrete extreme points. In other words, the result is as discrete as our assumptions allow. For example:

- when we assume that the reconstructed signal is monotonic, the reconstructed function is often (piece-wise) constant;
- if we additionally assume that the signal is one time differentiable, the result is usually one time differentiable, but rarely twice differentiable, etc.

#### 3.1.3. Tsirelson’s Explanation

Out of many papers that mention the puzzling discreteness, we cited [16]—because this paper not only *mentions* the fact of discreteness, it also provides an *explanation* for this discreteness, and this explanation is closely related to the Central Limit Theorem; see also [17].

Indeed, when we extract a signal from a mixture with Gaussian noise, then the *maximum likelihood* estimation (a traditional statistical technique; see, e.g., [2]) means that out of all possible signals from the given class of signals, we look for the signal which is the closest (in the least squares—i.e., in effect, Euclidean—metric) to the observed “signal + noise” combination.

In particular, if the signal is determined by finitely many (say, *d*) parameters, we must look for a signal $\vec{s}=\left(s_{1},\ldots ,s_{d}\right)$ from the a priori set $A\subseteq {I\;R}^{d}$ that is the closest (in the usual Euclidean sense) to the observed values

$$\vec{o}=\left(o_{1},\ldots ,o_{d}\right)=\left(s_{1}+n_{1},\ldots ,s_{d}+n_{d}\right),$$

where $n_{i}$ denotes the (unknown) values of the noise.

Since the noise is Gaussian, we can conclude that the average value of ${\left(n_{i}\right)}^{2}$ is close to $\sigma^{2}$, where σ is the standard deviation of the noise. In other words, we can conclude that

$${\left(n_{1}\right)}^{2}+\ldots +{\left(n_{d}\right)}^{2}\approx d\cdot \sigma^{2}.$$

In geometric terms, this means that the distance

$$\sqrt{\sum_{i=1}^{d}{\left(o_{i}-s_{i}\right)}^{2}}=\sqrt{\sum_{i=1}^{d}n_{i}^{2}}$$

between $\vec{s}$ and $\vec{o}$ is $\approx \sigma \cdot \sqrt{d}$. Let us denote this distance $\sigma \cdot \sqrt{d}$ by ε.

For simplicity of explanation, let us consider the case when d=2, and when *A* is a convex polygon. When the point $\vec{o}$ corresponding to observations is itself inside the set *A*, then this point is its own closest point in the set *A*. Let us consider the case when the point $\vec{o}$ is outside the set *A*. We can divide all points $\vec{o}$ which are outside the set *A* and which are ε-close to *A* into several zones depending on what part of *A* is the closest to $\vec{o}$: one of the *sides* (1D faces), or one of the *vertices*.

Geometrically, the set of all points $\vec{0}$ for which the closest point a∈A belongs to the *side* *e* is bounded by the straight line segments orthogonal (perpendicular) to *e*. The total length of this set is therefore equal to the length of this particular side; hence, the total length of the set of all the points that are the closest to the sides is equal to the *perimeter* of the polygon. This total length thus does not depend on ε at all.

On the other hand, the overall length of the set of all the points $\vec{o}$ at the distance ε from *A* grows with the increase in ε; this length grows approximately as the circumference of a circle, i.e., as constant ·ε.

When ε increases, the (constant) perimeter of the polygon *A* is a vanishing part of the overall length. Hence, for large ε:

- the fraction of the points that are the closest to one of the sides tends to 0, while
- the fraction of the points $\vec{o}$ for which the *closest* point from the set *A* is one of *A*’s *vertices* tends to 1.

Thus, with high probability, the reconstructed signal corresponds to one of the vertices (extreme points) of the set *A*.

Similar arguments can be repeated for any dimension *d*. For the same noise level σ, when *d* increases, the distance $\varepsilon =\sigma \cdot \sqrt{d}$ also increases, and therefore, for large *d*, for “almost all” observed points $\vec{o}$, the reconstructed signal is one of the extreme points of the *a priori* set *A*.

Much less probable is that the reconstructed signal $\vec{s}$ belongs to the 1-dimensional face of the set *A*, even less probable that $\vec{s}$ belongs to a 2-D face, etc.

#### 3.1.4. Methodological Consequence

So, when the dimension increases, we have a clear example of blessing of dimensionality: instead of having to consider a continuum of possible states, we only have to deal with a much smaller discrete set of extreme points—vertices of the corresponding polyhedron.

So, all observed phenomena fall into a few clusters—exactly as we observe in many cases.

*Comment:* This idea helps even in the quantum case. Namely, in quantum physics, there is a known paradox formulated by Schroedinger himself (the author of the main equation of quantum physics): while in quantum physics, we can have a superposition of any two states, why do we never see a superposition of two macro-states, e.g., of the state in which a cat is alive and the state in which the same cat is dead? This is indeed a serious problem, and it was one of the reasons why Einstein did not believe that quantum physics was an adequate description of reality; see, e.g., [18,19,20].

Strictly speaking, this is not a paradox in the purely logical sense of the word—it is just a contradiction between our intuition and the predictions of quantum theory. Many features of quantum physics are counter-intuitive, but usually, such counter-intuitive features are about the micro-world of elementary particles, not about the usual macro-size objects. The above idea makes this contradiction less troubling, because it implies that with very high probability, we will observe one of the two original states and not their convex combination (i.e., in this case, not their superposition).

#### 3.1.5. Resulting Discreteness Is Only Approximate

Of course, as with every probabilistic phenomenon, the above conclusion about discreteness is only approximate: we do not necessarily get one of the vertices, we get a point which is *close* to one of the vertices. This is why we did not write that all observed phenomena *coincide* with one of the few cases—we wrote that all observed phenomena fall into a few *clusters*. Within each cluster, we still have continuous changes—e.g., we can have cats of different length, different weight, etc.

### 3.2. Uncertainty Quantification and Probabilistic Limit Theorems—Including Theorems beyond Normal Distributions

#### 3.2.1. Need for Data Processing

What are the main objectives of science and engineering? We want to *understand* the world, i.e., to learn the values of the quantities that characterized the current state of the world. We want to *predict* the future state of the world, i.e., we want to predict the future values of the corresponding quantities. Furthermore, finally, we want to *change* the world—we want to find the design parameters that satisfy given specifications, we want to find the control values that will lead a system to the desired state, etc.

Some quantities that describe the world we can directly measure: e.g., the distance between two houses on the same street. For many other quantities, we cannot measure them directly: e.g., the distance to a nearby star. Furthermore, we clearly cannot directly measure the future values of the quantities or the adequate value of control parameters. All these quantities have to be estimated based on the known information about the world, i.e., based on the results of measuring some measurable quantities.

To estimate a desired quantity *y*, we need to know the relation $y=f(x_{1},\ldots ,x_{n})$ between this quantity and measurable quantities $x_{1},\ldots ,x_{n}$. Sometimes, we know an explicit analytical expression for this relation. In many other cases, we just know an algorithm that computes *y* from the values $x_{i}$. This algorithm can include a numerical solution of a complex system of non-linear differential equations—as when we predict tomorrow’s weather. The algorithm can also be a neural network trained to estimate the desired value *y* based on the known values $x_{1},\ldots ,x_{n}$.

#### 3.2.2. Need for Uncertainty Quantification

Whether we use neural networks or other algorithms for data processing, the inputs to all these algorithms are real numbers. These real numbers usually come from measurements, and measurements are never absolutely accurate; see, e.g., [21]. There is always noise. As a result, the measurement results ${\tilde{x}}_{i}$ are, in general, somewhat different from the actual (unknown) values $x_{i}$ of the corresponding quantities, and the difference $\Delta x_{i}\overset{\mathrm{def}}{=}{\tilde{x}}_{i}-x_{i}$—known as *measurement error*—is, in general, different from 0. So, when we apply the data processing algorithm *f* to the measurement results, the algorithm’s output $\tilde{y}=f\left({\tilde{x}}_{1},\ldots ,{\tilde{x}}_{n}\right)$ is, in general, different from the value $y=f(x_{1},\ldots ,x_{n})$ that we would have obtained if we knew the actual values $x_{i}$.

In practice, it is important to know how close our estimate $\tilde{y}$ is to the desired value *y*, i.e., in other words, how big can the difference $\Delta y\overset{\mathrm{def}}{=}\tilde{y}-y$ be. For example, suppose that we are prospecting for oil, and our estimate $\tilde{y}$ for the amount of oil *y* in the given region is 150 million tons. Then, if the accuracy is ±10 million tons, this estimate is good news, and we can start exploiting this region. On the other hand, if it is 150±200, then maybe there is no oil at all, so before we invest a lot of money into digging deep wells, we better perform more measurements to make sure that this money will not be wasted.

Estimating Δy is one the most important aspects of *uncertainty quantification*.

#### 3.2.3. Possibility of Linearization

We are interested in estimating the quantity

$$\Delta y=f\left({\tilde{x}}_{1},\ldots ,{\tilde{x}}_{n}\right)-f\left(x_{1},\ldots ,x_{n}\right)=f\left({\tilde{x}}_{1},\ldots ,{\tilde{x}}_{n}\right)-f\left({\tilde{x}}_{1}-\Delta x_{1},\ldots ,{\tilde{x}}_{n}-\Delta x_{n}\right).$$

Measurements are usually reasonable accurate, so the measurement errors $\Delta x_{i}$ are relatively small. For small values $\Delta x_{i}$, their squares ${\left(\Delta x_{i}\right)}^{2}$ are much smaller than the values themselves—and can therefore be usually safely ignored. For example, if $\Delta x_{i}\approx 10\%$, then ${\left(\Delta x_{i}\right)}^{2}\approx 1\%\ll \Delta x_{i}$. Thus, a reasonable idea is to expand the above expression for Δy in Taylor series and ignore terms which are quadratic (or of higher order) in terms of the measurement errors $\Delta x_{i}$. As a result, we get a linear dependence:

$$\Delta y\approx \sum_{i=1}^{n}c_{i}\cdot \Delta x_{i},\mathrm{where}\;c_{i}\overset{\mathrm{def}}{=}\frac{\partial f}{\partial x_{i}}.$$

*Comment:* This linearization—replacing the generic dependence with a linear one—is a common idea in applications. Actually, it one of the main ideas in many applications; see, e.g., [22].

#### 3.2.4. Here, the Central Limit Theorem Can Help

Let us first consider an important case—typically described in textbooks—when we know the probability distribution of each measurement error $\Delta x_{i}$. Usually, each measuring instrument is *calibrated*—if it has a *bias*, i.e., if the mean value $E[\Delta x_{i}]$ of the measurement error is not 0, we simply subtract this mean value from all the measurement results and thus, reduce it to 0.

In many practical applications, the number *n* of inputs is large, and the role of each of these inputs is relatively small. For example, one of the important data when prospecting for oil is seismograms—several-times-a-second recordings of the seismic signal. There are thousands of the corresponding values, and the effect of each individual value of the result of data processing is indeed small. The measurement errors corresponding to different measurements are usually reasonably independent. Thus, we are under the condition of the Central Limit Theorem—so we can conclude that the desired estimation error Δy is normally distributed.

A normal distribution is uniquely determined by its mean μ and its standard deviation σ. When each measurement error $\Delta x_{i}$ has mean value 0, the mean value of their linear combination Δy is also 0, and the variance σ of this linear combination can be determined from the known fact that the variance of the sum of independent random variables is equal to the sum of variances:

$$\sigma^{2}=\sum_{i=1}^{n}c_{i}^{2}\cdot \sigma_{i}^{2}.$$

#### 3.2.5. How Can We Actually Estimate σ?

In principle, we can directly use the above formula to estimate the standard deviation σ of the approximation error Δy. The main computational difficulty is that the data processing algorithm *f* is usually very complicated (especially in the case of neural networks), so it is not possible to compute the partial derivatives analytically. We can, however, use the fact that a partial derivative is defined as the limit of the ratios

$$\frac{\partial f}{\partial x_{i}}=\lim_{h\to 0}\frac{f\left({\tilde{x}}_{1},\ldots ,{\tilde{x}}_{i-1},{\tilde{x}}_{i}+h,{\tilde{x}}_{i+1},\ldots ,{\tilde{x}}_{n}\right)-\tilde{y}}{h},$$

and thus, for a sufficiently small *h*, the value of the ratio is very close to the desired partial derivative. Thus, we can estimate $c_{i}$ as

$$c_{i}\approx \frac{f\left({\tilde{x}}_{1},\ldots ,{\tilde{x}}_{i-1},{\tilde{x}}_{i}+h,{\tilde{x}}_{i+1},\ldots ,{\tilde{x}}_{n}\right)-\tilde{y}}{h}.$$

The problem with this idea is that it takes too long. Indeed, if we have several thousand inputs, then, to compute all the corresponding values $c_{i}$, we need to call the data processing algorithm *f* (which often takes hours to compute) n+1 times: one time to compute $\tilde{y}$, and *n* time to compute the corresponding *n* ratios $c_{i}$. For several thousand inputs, this is not realistic.

The good news is that we can instead use Monte-Carlo techniques: instead of computing *n* partial derivatives, we can simply emulate certain number of times *K*, measurement errors $\delta x_{i}^{\left(k\right)}$ which are normally distributed with standard deviation $\sigma_{i}$, and compute the differences

$$\delta y^{\left(k\right)}=\tilde{y}-f\left({\tilde{x}}_{1}-\delta x_{1}^{\left(k\right)},\ldots ,{\tilde{x}}_{n}-\delta x_{n}^{\left(k\right)}\right).$$

By the same logic as before, the differences $\delta y^{\left(k\right)}$ are normally distributed with the desired standard deviation σ. Thus, from a sample of *K* values, we can estimate σ with accuracy $\approx 1/\sqrt{K}$ [2]. So, if we want to estimate σ with relative accuracy $1/\sqrt{K}\approx 20\%$, it is sufficient to call the algorithm *f* K=25 times—which is much smaller than thousands needed for exact estimation.

#### 3.2.6. So What?

Why are we spending so much time on ideas that are well known to many readers? Because this will prepare readers for something that—unfortunately—not too many readers know: that we can use limit theorems beyond normal distributions to cover other realistic cases of uncertainty quantification.

#### 3.2.7. Need for Interval Uncertainty

In the previous text, we assumed that for each measurement, we know the probability distribution of the corresponding measurement error. The usual way to find this distribution is to *calibrate* the given measuring instrument (MI), i.e., to compare its results with the results of a “standard” (=much more accurate) measuring instrument. Since the standard measuring instrument (SMI) is much more accurate than the one we are calibrating, we can safely ignore SMI’s measurement errors (in comparison with MI’s measurement errors), and take the results measured by SMI as true values.

However, there are two important cases when calibration is not done. The first is the case of state-of-the-art measurements, when the MI that we have is the best there is. It would be great if near the Hubble telescope, there would fly a five times more accurate instrument for measuring the stars’ locations, but this telescope is the best we have. Similarly, in geophysics, oil prospecting companies use the best measuring instruments they can find—these instruments are expensive, but digging a well in the location where there is no oil would be much more expensive. In this case, there is no SMI to compare, so we cannot calibrate our MI.

Another case is manufacturing and other practical applications. In this case, in principle, we can calibrate every single measuring instrument and determine its probability distribution. However, nowadays, many sensors are cheap—e.g., kids playing with robots buy distance sensors for a few bucks. However, calibrating a sensor means utilizing a standard measuring instrument, which is usually much more expensive to use. The companies usually cannot afford to calibrate all their sensors. Instead, we have to rely on the information provided by the manufacturers of the corresponding measuring instruments.

The manufacturer of the MI also has the option to calibrate it—but since this calibration costs a lot, the calibrated sensors, with certified probability distributions of measurement errors, cost much more. It is much cheaper to buy a sensor for which only the minimum of necessary information is provided. In practice, this means that the only information that we have about the measurement error Δx is an upper bound Δ on its absolute value: |Δx|≤Δ. (At least such an upper bound needs to be provided—otherwise, it is not a measuring instrument, it is a wild guess.)

Once we know the upper bound $\Delta_{i}$ on the absolute value $|\Delta x_{i}\left|=\right|{\tilde{x}}_{i}-x_{i}|$ of each measurement error, then, based on the measurement result ${\tilde{x}}_{i}$, the only information we gain about the actual (unknown) value $x_{i}$ of the corresponding quantity is that this value belongs to the interval $\left[{\underline{x}}_{i},{\bar{x}}_{i}\right]\overset{\mathrm{def}}{=}\left[{\tilde{x}}_{i}-\Delta_{i},{\tilde{x}}_{i}+\Delta_{i}\right]$. Because of this fact, such a situation is known as *interval uncertainty*.

#### 3.2.8. Is the Corresponding Distribution Gaussian?

If we carefully eliminated all major sources of measurement error, then only small factors remain that affect the measurement error. Thus, due to the Central Limit Theorem, we can safely conclude that the distribution of the measurement error is close to Gaussian. Will that help? Not really: since we did not do the calibration, we do not know what is the bias. In principle, the bias can take any value from $-\Delta_{i}$ and $\Delta_{i}$, so the fact that we have a normal distribution will not decrease the interval of uncertainty.

#### 3.2.9. Uncertainty Quantification: Case of Interval Uncertainty

Under interval uncertainty, the only thing we can conclude about the value $y=f(x_{1},\ldots ,x_{n})$ that we would have obtained if we used the actual (unknown) values of the quantities $x_{i}$ is that it belongs to the *range*$\left[\underline{y},\bar{y}\right]$ of possible values of the function *f* when $x_{i}$ are in the corresponding intervals:

$$\left[\underline{y},\bar{y}\right]=\left\{f\left(x_{1},\ldots ,x_{n}\right):x_{i}\in \left[{\underline{x}}_{i},{\bar{x}}_{i}\right]\mathrm{for}\;\mathrm{all}\;i\right\}.$$

The problem of computing this interval is known as the problem of *interval computation*; see, e.g., [23,24].

In general, this problem is NP-hard [25]—which means that, unless P = NP (which most computer scientists do not believe to be possible), no feasible algorithm is possible for solving all particular cases of this problem. However, in the linearized case, a feasible algorithm *is* possible. Indeed, since the expression $\sum_{i}c_{i}\cdot \Delta x_{i}$ is linear (thus monotonic) in the variables $\Delta x_{i}$, its largest value is attained:

- for $c_{i}>0$, when the value $\Delta x_{i}$ is the largest, i.e., when $\Delta x_{i}=\Delta_{i}$, and
- for $c_{i}<0$, when the value $\Delta x_{i}$ is the smallest, i.e., when $\Delta x_{i}=-\Delta_{i}$.

Thus, the largest possible value Δ of Δy is equal to

$$\Delta =\sum_{i=1}^{n}\left|c_{i}\right|\cdot \Delta_{i}.$$

Similarly, one can easily show that the smallest possible value of Δy is equal to −Δ.

#### 3.2.10. How to Estimate Uncertainty in the Interval Case

How can we compute this sum Δ? We can directly use this formula, i.e., use numerical differentiation to compute all the partial derivatives $c_{i}$ and then compute the sum. However, as we have mentioned earlier, in many practical situations, this approach is not realistic. What can we do?

#### 3.2.11. Another Limit Distribution Comes to the Rescue

As we have mentioned, the convergence to a normal distribution only happens under certain conditions. In other cases, we may have convergence to other so-called *infinitely divisible* distributions [2]. One of such distributions is the *Cauchy distribution*, in which the probability density ρ(x) has the following form:

$$\rho \left(x\right)=\mathrm{const}\cdot \frac{1}{1+{\left(\frac{x}{\Delta }\right)}^{2}},$$

for some parameter Δ.

An important feature of the Cauchy distribution is that if we have several independent Cauchy distributed random variables $r_{i}$ with parameters $\Delta_{i}$, then their linear combination $\sum_{i}c_{i}\cdot r_{i}$ is also Cauchy distributed, with parameter $\Delta =\sum_{i}\left|c_{i}\right|\cdot \Delta_{i}$—which is exactly the value that we want to compute. This feature leads to the following Monte-Carlo method for computing Δ: we emulate a certain number of times *K*, measurement errors $\delta x_{i}^{\left(k\right)}$ which are Cauchy distributed with parameters $\Delta_{i}$, and compute the differences

$$\delta y^{\left(k\right)}=\tilde{y}-f\left({\tilde{x}}_{1}-\delta x_{1}^{\left(k\right)},\ldots ,{\tilde{x}}_{n}-\delta x_{n}^{\left(k\right)}\right).$$

Then, due to the above feature, the differences $\delta y^{\left(k\right)}$ are Cauchy distributed with the desired parameter Δ. Thus, to a sample of *K* values, we can apply, e.g., the maximum likelihood method [2], and thus estimate Δ with accuracy $\approx 1/\sqrt{K}$. Similarly to the case of normal distributions, this drastically speeds up computations: if we want to estimate Δ with relative accuracy 20%, it is sufficient to call the algorithm *f* 25 times—which is much smaller than thousands of times needed for exact estimation.

This method has been successfully used in many applications; see, e.g., [26].

*Comment:* Note that, in contrast to many simulation techniques, the use of Cauchy distribution in interval-related uncertainty quantification is *not* a realistic simulation:

- the actual measurement error is always located *inside* the interval [−Δ,Δ], while
- the Cauchy-distributed random variable has a non-zero probability to be anywhere, in particular, *outside* the interval.

### 3.3. What If We Have No Information about Probabilities

#### 3.3.1. Formulation of the Problem

What if we know that the disturbance $x=(x_{1},\ldots ,x_{n})$ is a joint effect of several independent small ones: $x=x^{\left(1\right)}+\ldots +x^{\left(N\right)}$, where about each component $x^{\left(i\right)}$, we only know the set $X^{\left(i\right)}$ of its possible values—and we do not have any information about probabilities of different points within each set. The only constraint is that all the points from each set $X^{\left(i\right)}$ are small, i.e., that for some small values ε>0, the length $∥x^{\left(i\right)}∥$ of each vector $x^{\left(i)\right)}\in X^{\left(i\right)}$ does not exceed ε. We will call such sets *ε-small*.

In this case, the set *X* of all possible values of the sum *x* is the set of all possible sums $x^{\left(1\right)}+\ldots +x^{\left(N\right)}$, where $x^{\left(i\right)}\in X^{\left(i\right)}$ for all *i*. In mathematics, the set of all such sums is known as the *Minkowski sum* of the sets $X^{\left(i\right)}$. The Minkowski sum is usually denoted by $X^{\left(1\right)}+\ldots +X^{\left(N\right)}$.

What can we say about such set *X*?

#### 3.3.2. 1-D Case

The 1-D case n=1 was studied in [27]. This paper showed that if a set *X* is the Minkowski sum of several ε-small closed sets, then it is ε-close to some interval I=[a,b], i.e.,:

- every point from the set *X* is ε-close to some point from the interval *I*; and
- every point from the interval *I* is ε-close to some point from the set *X*.

In the limit ε→0, we conclude that the Minkowski sum tends to the interval.

To be more precise, the following results were proven:

**Theorem** **1.** *If a set S⊆IR is a Minkowski sum of δ-small closed sets, then S is δ-close to an interval.*

**Theorem** **2.** *If a set S⊆IR can be, for every δ>0, represented as a Minkowski sum of finitely many δ-small closed sets, then S is an interval.*

*Comment:* This limit theorem is similar, in formulation, to the Central Limit Theorem and its generalizations: it shows that if a quantity can be represented as the sum of many small components, then the set of all possible values of this quantity is close to an interval—and the smaller the components, the closer is the resulting set to an interval.

Similarly to the fact that the original Central Limit Theorem explains the real-life ubiquity of normal distributions, this limit theorem explains the ubiquity of interval uncertainty; see, e.g., [21,23,24].

#### 3.3.3. General Case

It is well known that every convex set *X* containing 0 can be represented, for every ε>0, as a Minkowski sum of ε-small sets: indeed, it is sufficient to take $X^{\left(i\right)}=N^{-1}\cdot X$ for a sufficiently large *N*, then:

- The inclusion $X\subseteq X^{\left(1\right)}+\ldots +X^{\left(N\right)}$ follows from the fact that each element *x* can be represented as the sum $x=N^{-1}\cdot x+\ldots +N^{-1}\cdot x$; and
- the opposite inclusion $X^{\left(1\right)}+\ldots +X^{\left(N\right)}\subseteq X$ follows from the fact that the set *X* is convex and thus, once the elements $x^{\left(1\right)},\ldots ,x^{\left(N\right)}$ belong to this set, their convex combination $N^{-1}\cdot x^{\left(1\right)}+\ldots +N^{-1}\cdot x^{\left(N\right)}$ also belongs to *X*.

Whether the opposite is true, i.e., whether only convex sets can be represented as sums of small sets, remained an open problem. This problem—first formulated in [27]—was resolved in [28], where the following result was proven:

**Theorem** **3.** *If a set $X\subseteq {I\;R}^{n}$ can be represented, for each ε>0, as a Minkowski sum of ε-small closed sets, then this set X is convex.*

To be more precise, this paper proved the following result:

**Theorem** **4.** *For every γ>0, if a set $X\subset {I\;R}^{n}$ of diameter <1 is δ-close to a Minkowski sum of sets of diameter ≤ε, then X is γ-close to a convex set, for δ=γ/3 and $\varepsilon =\gamma^{2}/\left(20n\right).$*

*Comment:* This limit theorem explains the ubiquity of convex set in real-life problems. This is very good news, since it is known that convexity makes many computational problems easier to solve; see, e.g., [29].

### 3.4. Important Open Questions

#### 3.4.1. What if We Only Have Partial Information About Probabilities?

In the above, we first considered cases where we know the probability distributions of the aggregated factors before moving to those in which when we only know the ranges, and we have no information about the probability of different values from these ranges. These are two extreme situations—either we know everything about the probabilities, or we have no information about these probabilities at all. In practice, we often have intermediate situations, when we have *partial* information about the probabilities. It is therefore desirable to extend the limit results from both extreme cases to the such intermediate situations as well.

#### 3.4.2. Possible Approach and Natural Generalizations of the Central Limit Theorem

When we know all the probabilities, then for uncertainty quantification, we can use Monte-Carlo approach with normal distributions. When we only know the upper bounds, we can use Cauchy distributions. What if for some components, we know the probabilities, and for others, we only know bounds? The resulting random variable is the sum of two partial sums, for which the first partial sum can be handled by the normal distribution, while the second partial sum can be handled by the Cauchy distribution. In this case, it seems reasonable to use the distributions corresponding to the sum of normally and Cauchy distributed random variables.

The family of such distributions is also a natural limit—the limit of sums in which the first partial sum tends to normal distribution and the second partial sum tends to the Cauchy one. Such mixed distributions are not covered by the usual limit theorems, which only consider 2-parametric limit families of probability distributions: e.g., a normal distribution is determined by two parameters—the mean and standard deviation of the normal distribution. Sums would require more parameters: we need mean and standard deviation of the normal part and the parameter Δ of the Cauchy part.

Possible generalizations of the traditional limit theorems to such multi-parametric families have been analyzed in [30]. It turns out that, in general, in this case, the resulting distribution is equivalent to the distribution of the sum of several different infinitely divisible distributions: e.g., to the sum of normally and Cauchy distributed variables. So maybe other distributions of this type can be used for uncertainty quantification in other cases when we only have partial information about probabilities?

#### 3.4.3. What if We Are Interested in the Extreme Case?

Very often, we are interested in the extreme case: e.g., when we design a bridge, we want it to withstand the strongest possible winds that can happen in this area. In such situations, we are interested not in the summary effect of several random variables, but rather in the largest value $x=\max(x_{1},\ldots ,x_{n})$ of several random variables $x_{i}$—e.g., variables describing the wind on different days. When all these variables are identically distributed, then, similarly to the Central Limit Theorem, we have a finite-parametric family of distributions that represents the distribution of such extreme events; see, e.g., [31,32,33,34,35,36,37,38]. Such results are known as *Extreme Value Theory*. The most widely used result is that if the random variables $x_{i}$ are independent and identically distributed, then, under reasonable conditions, as *n* increases, the cumulative distribution function of the maximum *x* of these variables tends to one of the three distribution functions: Gumbel law

$$F\left(x\right)=\exp\left(-\exp\left(-\frac{x-b}{a}\right)\right),$$

Fréchet law

$$F\left(x\right)=\exp\left(-{\left(\frac{x-b}{a}\right)}^{-\alpha }\right)\mathrm{for}\;x>b,$$

and Weibull law

$$F\left(x\right)=\exp\left(-{\left|\frac{x-b}{a}\right|}^{\alpha }\right)\mathrm{for}\;x<b.$$

This result is actively used in practice, e.g., in reliability engineering, to estimate the probability of an extreme event.

The above result holds when all the variables $x_{i}$ are identically distributed. In reality, the distributions of the corresponding values $x_{i}$ are, in general, somewhat different. So, a natural question is: can we extend the Extreme Value Theory to such more general case? A similar generalization is possible for the Central Limit Theorem: it holds for the sum $x=x_{1}+\ldots +x_{n}$ even when the distributions of different variables $x_{i}$ are different. However, no such extension is known for the Extreme Value Theory. The absence of such general extension is not caused by our inability to prove the corresponding result: it can be shown that, if we simply remove the restriction that all variables $x_{i}$ are identically distributed, then the set of all limit distributions is no longer finite-dimensional; see [39].

Due to the practical importance of the Extreme Value Theory, an important question emerges: since in a *general* case, we have an infinite-dimensional family of limit distributions, can we find *specific* cases when distributions are different, but a finite-dimensional family of limit distributions is still possible?

## 4. Dimensionality of Temporal Origin

### 4.1. Case Study

Let us consider the case of a simple hardware sensor, in which the input *x*—e.g., intensity of light—generates a signal that goes through multiple layers until it produces the final electric signal. When passing through these layers, the signal undergoes a sequence of transformations. These transformations are, in general, nonlinear. In mathematical terms, this means that the resulting transformation f(x) of the original real value *x* to the 1D sensor output f(x) is a composition of several different nonlinear functions

$$f\left(x\right)=f_{n}\left(f_{n-1}\left(\ldots f_{2}\left(f_{1}\left(x\right)\right)\ldots \right)\right).$$

We can consider the sensor as a whole, with the transformation function f(x). We can divide it into several layers and consider the overall value-to-signal transformation f(x) as a composition of transformations corresponding to different layers. Each of these layers can be viewed as several sub-layers, so the corresponding value *n* can be very large—and transformations $f_{i}\left(x\right)$ corresponding to all these very thin sub-layers are close to identity $f_{i}\left(x\right)\approx x$.

In the Central Limit Theorem, we took into account that the random variable *x* is equal to the sum $x=x_{1}+\ldots +x_{n}$ of a large number of small independent random variables, and we used the fact that under reasonable conditions, in the limit when n→∞, the distribution of this sum tends to a distribution from a known finite-parametric family—namely, to a normal distribution. The limit means that when *n* is large, the distribution of the sum *x* is close to Gaussian.

In our case, we consider a composition of a large number *n* of functions $f_{i}\left(x\right)$, which are close to identity. It is reasonable to look for situations in which, under some conditions, when *n* increases, such compositions would also tend to functions from some finite-parametric family. How can we describe the corresponding limit functions?

### 4.2. Let Us Formulate This Idea in Precise Terms

As we have mentioned earlier, in this paper, we do not focus on *conditions* when there is a convergence, we only focus on the resulting limit. In line with this approach, let us assume that we have a finite-parametric family *F* of limit functions.

If we have two sequences of transformations:

- A sequence $f_{i}$ whose compositions tend to some function f∈F; and
- a sequence $g_{i}$ whose composition tends to some function g∈F,

then in the case when we first apply all $f_{i}$-transformations and then all $g_{i}$-transformations, then the resulting limit function g(f(x)) should also belong to the family *F*. Thus, the desired family *F* of all possible limit functions should be closed under composition.

Most transformations in sensors are reversible. So, if we limit ourselves to such transformations, and instead of first applying $f_{1}$, then $f_{2}$, etc., we change the direction of signal processing and first apply $f_{n}^{-1}$, then $f_{n-1}^{-1}$, etc., then, in the limit, instead of the original limit function *f* we will get the inverse function $f^{-1}\left(x\right)$. So, the class *F* of all possible limit functions should contain, with each function *f*, its inverse function as well. So, the class *F* must be closed under composition and inverse. Such classes are known as *transformation groups*.

Furthermore, linear transformations are ubiquitous. Thus, it make sense to consider finite-parametric groups that contain all linear transformations. What are these groups?

### 4.3. Enter Norbert Wiener

Interestingly, the answer to this question is related to Norbert Wiener, the father of cybernetics. As he describes in his pioneering monograph [40] on cybernetics, when he started working on engineering problems, at first, he trusted exact mathematical models much more than vague biological analogies. Furthermore, then, when he came up with a draft design of a system for automatic vision, a neurophysiologist colleague Arturo Rosenblueth—who saw the corresponding picture—asked him with surprise since when Wiener had become interested in human vision: because it turned out that what Wiener came up with after many thoughts and tries was exactly the scheme implemented in human vision. This experience lead to Wiener’s idea of *cybernetics*, a science studying both engineering and biological systems, in which one of the main ideas is that since we humans are the product of billion years of improving evolution, our biology should be close to optimal—and thus simulating this biology can be very helpful in engineering.

In some cases, this optimality was indeed confirmed. In some other cases, Wiener became so confident in the related optimality that he made several mathematical hypotheses based on this confidence. For example, he learned, from Dr. Rosenblueth, that when we get closer and closer to an object, there are several clearly distinct phases in our visual perception (which, by the way, again fits with the above explanation of discreteness):

- When the object is very far, all we see is a formless blur—in other words, objects obtained from one another by arbitrary smooth transformations cannot be distinguished.
- When the object gets closer, we can detect whether it is smooth or has sharp angles. We may see a circle as an ellipse, or a square as a rhombus (diamond). At this stage, images obtained by a projective transformation are indistinguishable.
- When the object gets even closer, we can detect which lines are parallel, but we may not yet detect the angles. For example, we are not sure whether what we see is a rectangle or a parallelogram. This stage corresponds to affine transformation.
- Then, we have a stage of similarity transformations—when we detect the shape, but cannot yet detect its size.
- Finally, when the object is close enough, we can detect both its shape and its size.

Each stage can be thus described by an appropriate transformation group. So, Wiener conjectured that if there was a group intermediate between, e.g., all projective and all continuous transformations, our vision mechanism—the result of millions of years of improving evolution—would have used it. Thus, he formulated a hypothesis that such intermediate transformation groups are not possible [40].

Many mathematicians did not take this hypothesis too seriously—while they appreciated Wiener’s engineering ideas, they thought that he was going too far in his analogies. However, other mathematicians took it seriously—and, two decades after the first edition of Wiener’s book, they came up with a formal proof that, indeed, under reasonable conditions, there is only one transformation group that contains all linear (=affine) transformations and some non-linear ones: namely, the group of all projective transformations [41,42].

The general proof is very complicated, e.g., the paper [42] consists of more than 100 pages of dense mathematics. However, good news is that at present, we are only interested in the transformations of 1D signals. In this case, projective transformations are nothing else but fractional-linear ones

$$f\left(x\right)=\frac{a\cdot x+b}{c\cdot x+d},$$

and the corresponding proof can be shortened to a few pages; see, e.g., [43,44].

So, we arrive at the following conclusion.

### 4.4. So, What Are the Limit Transformations?

We have shown that limit transformations form a finite-parametric transformation group that contains all linear transformations, and that all transformations from such a group are fractional linear—with linear ones being a particular case.

Thus, we conclude that all limit transformations are fractional-linear.

### 4.5. A Similar Conclusion Can Be Made about All Possible Reasonable Transformations

Instead of looking for *limit* transformations, we can consider a different problem: to describe a class of all transformations which are, in some sense, *reasonable*. Linear transformations are reasonable: shift corresponds to changing the starting point and a multiplication by a number corresponds to changing a measuring unit. A good example of both transformations are transformation between Celsius and Fahrenheit temperature scales.

It is also natural to conclude that a composition of two reasonable transformations is reasonable, and that a transformation which is inverse to a reasonable transformation is also reasonable. If we want to use computers to deal with reasonable transformations, it also makes sense to require that the reasonable transformations form a finite-parametric family—since in a computer, we can only stored finitely many parameter values.

Thus, the class of all reasonable transformations forms a finite-parametric transformation group containing all linear transformations. So, we conclude that every reasonable transformation is fractional linear.

### 4.6. What Are the Implications for Neural Networks?

Artificial neural networks—a perfect example of Wiener’s belief that emulating biological systems can be beneficial—are formed of *neurons*. In a neuron, first, we form a linear combination *x* of the inputs $x_{i}$, and then we apply some non-linear transformation y=s(x) to this linear combination. In neural networks, this nonlinear transformation is known as an *activation function*.

Which activation function should we use? The first nonlinear neurons use *sigmoid* activation function

$$s\left(x\right)=\frac{1}{1+\exp(-x)},$$

because, in the first approximation, this is how signals are processed in biological neurons; see, e.g., [14]. This activation function worked very well—much better than other activation functions that have been tried. This activation function is still often used in some layers of deep neural networks [15], where they are also very successful. How can we explain this success?

A possible explanation comes from the fact that, as we have mentioned earlier, all inputs come with noise. The simplest case is when, for each measurement, we just have a constant noise $n_{i}=\mathrm{const}$, when instead of the actual values $x_{i}$, the measurement results are shifted by this value $n_{i}$, to $x_{i}+n_{i}$. As a result, the linear combination *x* is also shifted by some constant *n* (which is the similar linear combination of noises $n_{i}$):

x→x+n.

We do not know the exact value of this noise—if we knew, we could simply subtract it from all the measured values. It is therefore reasonable to require that the result of applying the activation functions should be insensitive to this noise as much as possible.

Of course, we cannot simply require that s(x+n)=s(x) for all *x* and *n*—this would imply that the function s(x) is a constant that does not depend on the input at all. This makes sense: for example, the formula d=v·t showing that the distance can be obtained by multiplying velocity and time does not change when we change the unit of time, e.g., from hours to seconds. However, this invariance does not mean that the formula remains exactly the same when we change the unit of time: to keep the formula the same, we also need to apply an appropriate transformation to velocity as well: namely, replace the values in km/h with a value in km/sec. Similarly here, a natural idea is to require that if we apply a shift $x\to x^{\prime }=x+n$ to the input, the formula remains the same if we apply an appropriate transformation to *y* as well, i.e., that $y^{\prime }=s\left(x^{\prime }\right)$, where $y^{\prime }=T\left(y\right)$ for some reasonable transformation *T*.

In other words, we conclude that for every value *n*, there exists some reasonable transformation $T_{n}$ for which $s\left(x^{\prime }\right)=T_{n}\left(y\right)$. Here, $x^{\prime }=x+n$, and y=s(x), so $s\left(x+n\right)=T_{n}\left(s\left(x\right)\right)$. We have already concluded that reasonable transformations are fractional linear, thus we have

$$s\left(x+n\right)=\frac{a(n)\cdot x+b(n)}{c(n)\cdot x+d(n)}$$

for some values a(n) through d(n). To describe all the functions s(x) that have this property, we can differentiate both side of this equation by *n* and take n=0. The resulting differential equation can then be explicitly solved; see, e.g., [43,45,46]. The generic monotonic solution to this equation indeed differs from the sigmoid activation functions only by linear transformations of *x* and *y*.

This explains why the sigmoid activation function indeed works well in *many* application problems.

*Comment:* Of course, this does not mean that this activation function works best in *all* practical applications. For example, in most layers of deep neural networks, a different activation function s(x)=max(0,x)—known as *rectified linear* activation function—works much better. Interestingly, similar invariance ideas can explain the use of the rectified linear activation function—as well many other empirically successful features of deep learning algorithms; see, e.g., [46].

## 5. Conclusions

In this paper, we showed that limit theorems—similar to the Central Limit Theorem from statistics—make analysis of complex systems easier—i.e., lead to the blessing-of-dimensionality phenomenon. We showed that this simplification happens for all the aspects of these systems:

- For the corresponding transformations—as shown, e.g., by the description of all possible limit and/or reasonable transformations, and by the resulting theoretical explanation of the efficiency of sigmoid activation functions;
- for the system’s uncertainty—as shown, e.g., by the use of limit distributions such as normal and Cauchy to make uncertainty quantification more efficient, and by the use of limit theorems to explain the ubiquity of interval uncertainty; and
- the desired result of the system’s analysis—as shown, e.g., by a limit-theorem-based explanation of why it is usually possible to meaningfully classify objects into a small finite number of classes.

## Data Availability

Not applicable.
